# Quality Improvement Project on the Use of Zopiclone on Elm Ward, West Park Hospital

**DOI:** 10.1192/bjo.2026.11449

**Published:** 2026-06-30

**Authors:** Omeno Ohene, Obinna Anakwe

**Affiliations:** Tees, Esk and Wear Valley, Darlington, United Kingdom

## Abstract

**Aims:**

Zopiclone is licensed for the short-term management of insomnia, with recommended treatment durations of 2–5 days for transient insomnia, 2–3 weeks for short-term insomnia, and not exceeding four weeks including tapering, in line with NICE TA77 guidance. Elm Ward’s initial audit (Oct–Dec 2023) identified widespread prescribing beyond these limits. This re-audit provides updated patient-level data to assess progress following practice changes introduced after the initial audit.

The re-audit aimed to:

1. Evaluate current zopiclone prescribing patterns.

2. Compare durations of use with the 2023 audit.

3. Assess compliance with recommended maximum treatment duration.

4. Identify remaining gaps in documentation and review processes.

**Methods:**

The first audit included 48 patients, of whom 22 received zopiclone. The re-audit sampled 43 patients from Elm Ward (Dec 2025–Feb 2026), identifying 12 prescribed zopiclone. Data sources included Kardex, EPMA, and CITO. Variables collected included demographics, indication, dose, start/stop dates, duration, review documentation, and use of alternative or non-pharmacological interventions. Where stop dates were missing, an end date of 05/02/2026 (the re-audit completion date) was applied to allow duration calculation.

**Results:**

In 2023, the mean duration of zopiclone use was 7.7 weeks (median 8.3; range 0.2–16), with only 27% (6/22) within the recommended ≤4-week limit. In the re-audit, 12 of 43 patients (27.9%) were prescribed zopiclone, with a mean age of 40 years. Using the audit end-date assumption, the mean duration was 3.42 weeks (~24 days), the median 1.29 weeks (~9 days), and the range 0.14–20.29 weeks. Overall, 83.3% (10/12) were within the ≤4-week maximum, and 16.7% (2/12) exceeded it. Indications were predominantly short-term insomnia, though documentation was incomplete in some cases. When judged against indication-specific NICE time windows, 30% of evaluable cases met the appropriate duration. Three patients received alternative sleep medications, and no non-pharmacological sleep interventions were documented. Dose was recorded in 83.3% (10/12), all at 7.5 mg.

**Conclusion:**

The re-audit shows a substantial improvement in prescribing duration compared with 2023, with most patients now receiving treatment within the recommended four-week limit. However, missing stop-date documentation continues to affect duration calculations and contributes to misclassification of treatment length. Persistent gaps remain in documenting non-pharmacological interventions, indications, and review plans. Continued reinforcement of documentation standards, pharmacist-led review prompts, and a repeat audit in 12 months are recommended to ensure sustained improvement.

